# Minimally Invasive Approach For Extraforaminal Synovial Cyst L5-S1

**DOI:** 10.7759/cureus.362

**Published:** 2015-10-22

**Authors:** Jose Torres Campa-Santamarina, Sara Towne, Marjan Alimi, Rodrigo Navarro-Ramirez, Roger Härtl

**Affiliations:** 1 Neurocirugia, Centro Medico Asturias, Oviedo, España; 2 Department of Neurosurgery, Weill-Cornell/New York Presbyterian Hospital

**Keywords:** synovial cyst, extraforaminal, lumbar spine, minimally invasive surgery

## Abstract

Symptoms from synovial cysts are produced by neural compression in the spinal canal or the foramen. Few cases of extraforaminal synovial cyst have been published in the literature. This is a case report of a 65-year-old female who presented with a three-month history of sciatic pain and no relief with conservative treatment. MRI showed a left-sided extraforaminal synovial cyst at L5-S1 with compression of the L5 nerve root at the lateral portion of the foramen. Minimally invasive surgery for resection was performed using an extraforaminal tubular microscopic endoscopy-assisted approach. The patient improved clinically and remained symptom-free for the entire follow-up of 30 months.

## Introduction

Synovial cysts were first described in the spine in 1950 by Vosschulte and Borger, though they were documented in the knee as early as 1885 by Baker [1]. Spinal synovial cysts typically originate from degeneration of the facet joints, although a few cases resulting from microtrauma have also been published [2]. An extraforaminal location of the cyst is fairly uncommon; over 1,000 cases of a synovial cyst inside the spinal canal have been published, but to our knowledge, only 13 cases of extraforaminal synovial cyst have been reported [3-9].

Advances in MRI technology in recent years have facilitated diagnosis of synovial cysts. Cysts appear isointense on T1-weighted images and hyperintense on T2-weighted images (though hemorrhagic cysts are characterized by severe hypointensity on T2-MRIs). Coronal views are also useful for diagnosis and localization of the nerve root and evaluation of its relationship to the cyst.

Symptoms of synovial cyst consist of pain, numbness, and occasional paresis. The etiology of these symptoms stems from compression of the nerve root in the spinal canal. Compression occurs mainly in the lateral recess, where the canal is narrower. Increase in the volume of the cyst and irritation due to inflammation or bleeding can also result in pain along the dermatome of the compressed nerve.

Several instances of a synovial cyst with hemorrhagic features related to trauma and anticoagulation drugs have been published in the literature, although they comprise less than 10% of all cases [10]. The initial bleed can lead to an increase in the volume of the cyst and the inflammatory response around it, which can acutely exacerbate the symptoms caused by nerve root compression.

Although conservative management is the initial course of treatment for resolution of the symptoms, surgery happens to be the endpoint of many cases. Generally, open surgery involving resection of the facet joint and fusion is being performed. For two out of 13 cases of extraforaminal synovial cyst in the literature, a fusion was performed. All 13 required total or partial removal of the facet joint, typically indicating fusion, yet fusion was performed in only two cases out of all.

This case represents the first reported application of a minimally invasive surgical (MIS) technique to an extraforaminal synovial cyst. An endoscopy-assisted MIS approach allows for a reduction in the extent of facet joint removal. It lessens the degree of postoperative instability and consequently obviates the need for fusion.

## Case presentation

### History and examination

A 65-year-old female came to the hospital with a three-month history of sciatic pain with no lumbar complaints and no other major medical pathology. Her pain radiated through the left L5 dermatome, along with paresthesia extending into big toe. No previous trauma was recorded. No weakness was identified. Initial treatment was conservative management, including non-steroidal anti-inflammatory drugs, physical therapy, or morphine-derived drugs; however, no relief of the symptoms was achieved. She received two epidural injections of corticosteroids without any notable improvement.

Informed patient consent was obtained. No reference to the participant's identity was made at any stage in the paper. 

### Imaging studies

**Figure 1:**
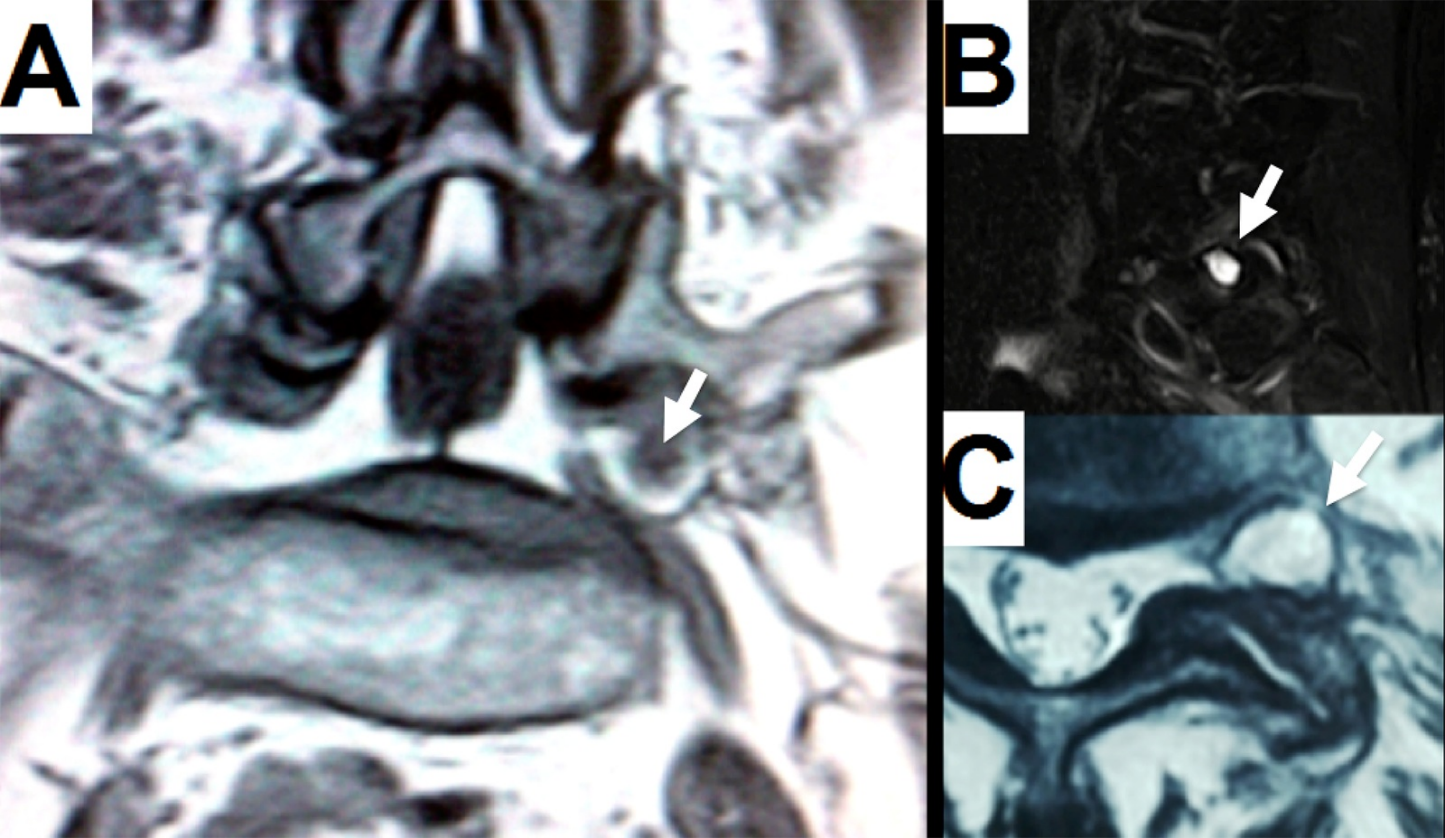
Preoperative MRI A: Coronal T2 shows a hyperintense cyst caudally displacing the root and compressing it with the sacral wing. Black content inside the cyst represents previous bleeding (white arrow). B: Sagittal T2 displays a hyperintense cyst on the left extraforaminal space (white arrow). C: Axial T2 shows hypertrophy of the facet joint into the left extraforaminal space (white arrow).

The lumbar magnetic resonance imaging (MRI) showed a left-sided extraforaminal synovial cyst at L5-S1 with compression of the L5 nerve root at the lateral portion of the foramen on the coronal projection (Figure 1A). On the sagittal projection, a hyperintense T2 image compatible with a high fluid content lesion was also seen (Figure 1B). Finally, the same hyperintense T2 lesion was identified and marked on the axial cut of the indexed level (Figure 1C). Flexion-extension lumbar x-rays showed no signs of instability.

### Operation

Minimally invasive surgery was performed via the Wiltse approach. The patient was sedated under general anesthesia and was placed prone on a Wilson frame. Fluoroscopy was used to localize the appropriate level. A 2.0 cm lateral incision was made 3.5 cm left to the midline. A tubular retractor was placed over the lateral aspect of the facet joint, avoiding the iliac crest. A Leica M525 OH4 (Leica Microsystems, Switzerland) operating microscope was used during the operation. The facet joint was identified and the lateral aspect was exposed. Muscles were detached and osteophytes were drilled off (Hillan XS Aesculap Braun, Center Valley) up to the attachment point of the intertransverse ligament. The sacral wing was identified, and the intertransverse ligament was removed piecemeal with rongeurs until the synovial cyst and the L5 nerve root were visible. The root was severely compressed and displaced caudally; inflammatory tissues surrounded the nerve root. The extent of initial dissection off the nerve root was limited by the volume of the cyst. Puncture of the cyst was performed to allow further dissection off the root and the surrounding tissues; a viscous reddish-black liquid was extracted with a needle. The cyst was attached to the lateral aspect of the facet joint capsule, just under the exposed facet. A 30º endoscope was inserted to confirm complete resection as well as decompression of the nerve root. Coagulation was performed at the attachment point with 45º angulated bipolar forceps. Resection of the pedicle of the cyst was achieved using a 2 mm Kerrison rongeur.

### Pathology

Histological appearance was a sheet of synovial cells surrounded by fibroconnective tissue. Neo-angiogenesis, signs of bleeding, and deposits of hemosiderin were also visible.

### Postoperative course

**Figure 2:**
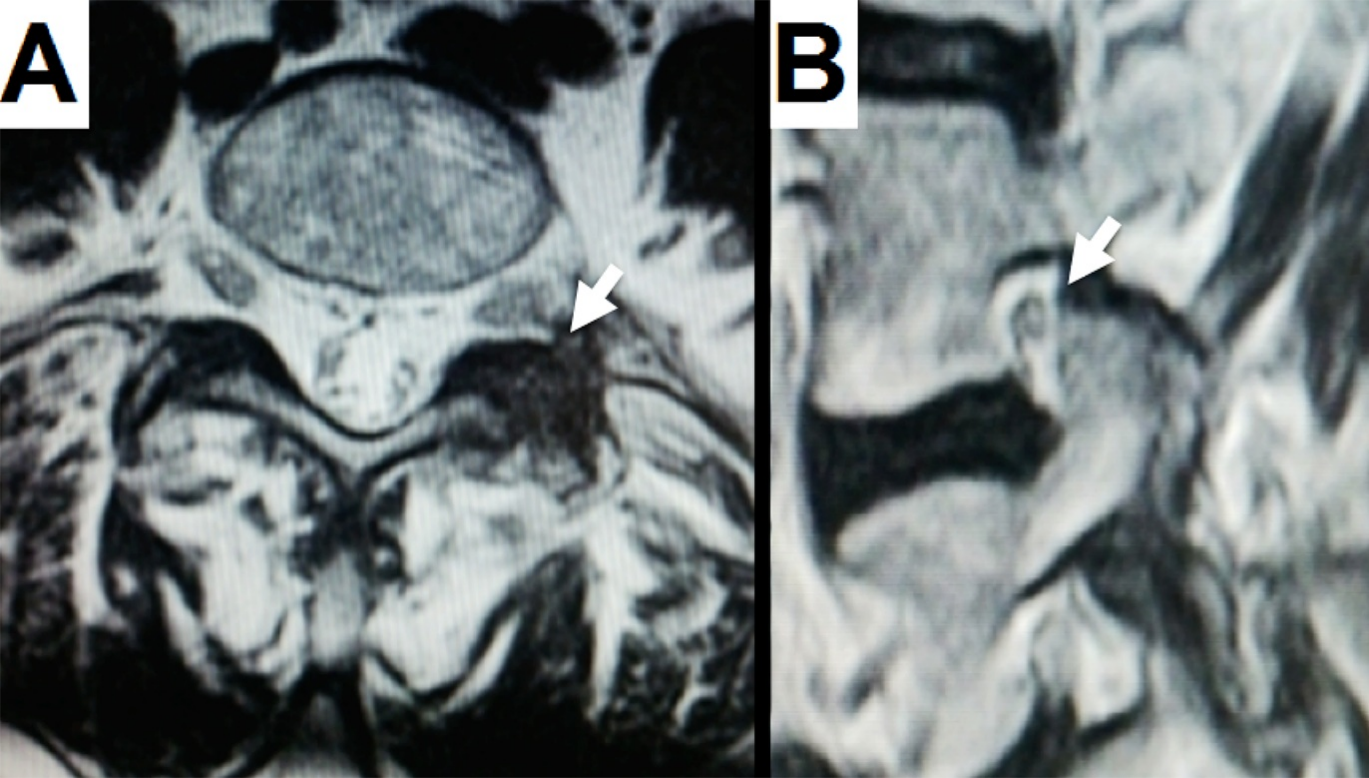
Postoperative MRI A: Axial T2 shows that the cyst has been completely removed (white arrow) and the lateral facet joint has been only minimally drilled off. B: Sagittal T2 indicates no scar tissue from the cyst behind the facet joint (white arrow).

The patient was completely symptom-free after surgery and was discharged after 12 hours. An MRI performed after three months showed no residual lesion (white arrows) on either the axial projection (Figure 2A) or the sagittal projection (Figure 2B). No sciatica, back pain, or other symptoms were reported during the three-year follow-up. 

## Discussion

Synovial cysts commonly arise from degeneration of the spinal facet joints and can be accompanied by degeneration-induced segmental instability. In treating synovial cysts, the primary aim is relief of the pain symptoms, while the secondary focus is laid on minimizing instability. Some authors suggest that patients with instability, detected on flexion-extension x-rays, need to receive fusion upfront.

Conservative management is the initial treatment of lumbar synovial cyst. Slipman, et al. reported 33% long-term pain relief with injections of corticosteroids [11]. Martha, et al. reported a 46% pain improvement with simultaneous injection and rupture of the cyst under fluoroscopy without using concomitant steroids [12]. Failure of conservative management indicates the need for surgery.

Open surgery is the conventional approach, often supplemented by fusion. Some authors recommend fusion as a standard protocol for all open surgeries of synovial cysts while others believe that it is required only in cases where preoperative flexion-extension x-rays show instability. Bydon, et al.’s meta-analysis of 966 patients who underwent either isolated excision (84%) or excision, plus fusion, found fusion not to be necessary in most cases [13]. The recurrence rate was 1.8% in isolated resection patients and 0% in resection, plus fusion cases. A second surgery for fusion was required in only 5.8% of the isolated excision cases, resulting in a total reoperation rate of 6.2%.

A fusion was documented in only two papers out of the 13 case reports on extraforaminal synovial cyst. Nonetheless, all thirteen surgeries involved significant resection of the facet joint, which usually would necessitate fusion [3-9].

An MIS approach to the synovial cyst was first published in 2004 by Sandhu, et al. [14] and his results were confirmed in 2006 by Sehati, et al. [15]. In 2012, James and Rhee published a contralateral MIS approach, allowing for better visualization of the cyst and less resection of the facet joints, which would result in less instability [16-17]. Altogether, the data support the value of MIS for resection of synovial cyst in the cases where preoperative instability is not present. In such cases where instability is present, joint facet preservation will not be necessary and subsequent segmental instrumentation would be recommended. 

An MIS approach to the synovial cyst can preclude the need for fusion; none of the 54 published cases required fusion during or after surgery. An MIS approach to synovial cyst has already been shown to reduce blood loss and muscle retraction and damage to the soft tissue, resulting in earlier ambulation and shorter hospital stays [18-23]. MIS surgery for herniated lumbar discs reduces muscle atrophy [20, 24]. These benefits are likely to be extended also to MIS treatment of extraforaminal synovial cysts. In addition, a tubular approach can preserve the majority of posterior elements, thereby minimizing the postoperative instability.

In the presented case, the Wiltse approach was successfully employed to gain access to the desired anatomy. As direct visualization of the anterior aspect of the facet is not possible with this approach, a 30º endoscope can be used. With minimal drilling and the help of an endoscope, the nerve root and normal anatomy can be identified. Also, puncturing or coagulation of the cyst wall can help to decrease the cyst size. These techniques allow for minimal damage to the facet joint and prevent the need for fusion. The data support a tubular approach to be a good treatment option for extraforaminal synovial cysts. Prior to surgery accessing the foramen via the Wiltse approach in a cadaver laboratory, it is important to understand the anatomical limitations, especially at the L5/S1 level where the iliac crest or the sacrum can represent an obstacle for this minimally invasive tubular approach. Although drilling off the crest is an option, it may complicate the surgery, and in many cases, open surgery should be performed instead.

Hemorrhagic cysts account for 9% of all synovial cysts; nonetheless, they do not significantly increase the complication rate. None of the published extraforaminal cases have reported hemorrhagic features [3-9]. The small chance of success with conservative management mandates early surgical treatment. The blood residue and inflammation surrounding the cyst may make dissection more difficult [10]. 

## Conclusions

Extraforaminal synovial cyst of the lumbar spine is a rare pathology, which can cause severe pain and is difficult to resolve with conservative management. The presented approach, involving a tubular retractor, microscope, and 30° endoscope-assisted procedure, reduces resection of the facet joint and thereby the concurrent risk of instability and subsequent need for fusion.
